# Phase-Ib dose-finding and pharmacokinetic trial of metformin combined with nivolumab for refractory/recurrent solid tumors

**DOI:** 10.1007/s10147-025-02786-2

**Published:** 2025-05-28

**Authors:** Toshio Kubo, Hironari Kato, Shigeru Horiguchi, Toshiyuki Kozuki, Akinori Asagi, Michihiro Yoshida, Heiichiro Udono, Katsuyuki Kiura, Katsuyuki Hotta

**Affiliations:** 1 Department of Allergy and Respiratory Medicine, Okayama University Hospital, Okayama, Japan; 2 Center for Clinical Oncology, Okayama University Hospital, Okayama, Japan; 3 Department of Gastroenterology, Okayama University Hospital, Okayama, Japan; 4 Department of Thoracic Oncology and Medicine, NHO Shikoku Cancer Center, Matsuyama, Japan; 5 Department of Gastrointestinal Medical Oncology, NHO Shikoku Cancer Center, Matsuyama, Japan; 6 Center for Innovative Clinical Medicine, Okayama University Hospital, Okayama, Japan; 7 Department of Immunology, Okayama University Graduate School of Medicine, Dentistry and Pharmaceutical Sciences, Okayama, Japan

**Keywords:** Pancreatic cancer, Thoracic tumors, Phase Ib, Anti-PD-1 antibody, Nivolumab, Metformin

## Abstract

**Background:**

Our previous findings showed that the addition of metformin to nivolumab resulted in remarkable tumor regression and increased the number of tumor-infiltrating T cells in mouse models. Therefore, we conducted a phase Ib study using combination therapy with nivolumab and metformin in patients with refractory/recurrent solid tumors.

**Methods:**

This study consisted of two parts: 1, evaluating the maximum tolerated dose (MTD), safety, pharmacokinetics in solid tumors, and 2, principally investigating the safety at the recommended dose limited to thoracic and pancreatic cancers. Metformin and nivolumab were administered orally at doses of 750–2,250 mg/day and biweekly at a fixed intravenous dose of 3 mg/kg, respectively. Dose-limiting toxicity was evaluated within the first 4 weeks. Both metformin and nivolumab were continued until disease progression or discontinued because of toxicity.

**Results:**

In total, 17 and 24 patients were enrolled in parts 1 and 2, respectively. One patient experienced increased pancreatic enzyme levels (grade 4) and lactic acidosis (grade 3). No Grade 5 adverse events were observed. MTD was not reached up to 2,250 mg/day of metformin, 2,250 mg/day was selected for part 2. An objective response was observed in 4 of 41 patients. One-year progression-free and overall survival rates were 9.8% and 56.8%, respectively. Two patients remained alive without disease progression for more than three years.

**Conclusions:**

Nivolumab and metformin combination therapy was well-tolerated and showed preliminary signals of efficacy in a subset of patients. Further verification of the underlying mechanism in cases where treatment is effective is required.

**Trial registration numbers:**

UMIN registration number 000028405 https://upload.umin.ac.jp/cgi-open-bin/ctr_e/ctr_view.cgi?recptno=R000031915.

**Supplementary Information:**

The online version contains supplementary material available at 10.1007/s10147-025-02786-2.

## Introduction

The development of immune checkpoint inhibitors (ICIs) has led to better survival outcomes, even for patients with advanced cancer [1–4]; however, such cases are rare. Nonetheless, various combinations of ICIs or cytotoxic anticancer agents have been tested to increase the number of patients who can benefit from them [5–7]. Recently, non-anticancer drugs, including statins and metformin have also been used in combination with ICIs to assess their potential in increasing anti-tumor efficacy of existing anticancer drugs [8–10].

We have long been interested in the synergistic effects of metformin and ICIs. Accordingly, we previously reported the remarkable regression of an engrafted tumor (RL male 1) with free drinking of metformin in a murine syngeneic model [11], which was not observed in T cell-deficient mice with severe combined immunodeficiency (SCID). With exposure to metformin, tumor-infiltrating CD8 T cells (CD8 TILs) changed from dominant central memory (TCM) to effector memory (TEM) cell types, leading to the acquisition of the polyfunctionality capable of producing multiple cytokines, such as IL-2, TNF-α, and IFN-γ [11]. Activation of glycolytic system in TEM may be linked with the enhanced polyfunctionality. The anti-PD-1 antibody also enhances the glycolytic system in T cells [12]. Thus, a combination of metformin and PD-1 blockade promotes infiltration and activation of CD8 T cells within tumor, resulting in tumor clearance [13, 14]. In another report, metformin decreases infiltration of FOXP3 T regulatory cells in intratumor regions, increases CD8 T cell infiltration in the peritumoral leading-edge stroma, and increases the CD8/FOXP3 ratio both in tumor and leading-edge stroma of primary head and neck squamous cell carcinoma [15]. There are also several clinical reports on the effect of metformin on the tumor immune environment [16, 17].

Based on these background data, we aimed to conduct a phase Ib study to evaluate the effect of combination therapy with nivolumab and metformin in refractory/recurrent solid tumors. We aimed to perform this study in two parts. Part 1 aimed to evaluate the safety of nivolumab metformin combination therapy in terms of maximum tolerated dose (MTD), dose-limiting toxicity (DLT), and adverse events, along with metformin pharmacokinetics. Effects of nivolumab metformin combination therapy in patients with non-small-cell lung cancer (NSCLC) or pancreatic cancer refractory to standard primary treatment were evaluated in Part 2.

## Patients and methods

### Eligibility

The eligibility criteria for part 1 were as follows: (1) pathologically confirmed solid tumor refractory to the standard treatment, (2) Eastern Cooperative Oncology Group performance status (ECOG PS) of 0 or 1, (3) age ≥ 20 years, (4) adequate organ function, (5) written informed consent from each patient, and (6) any evaluable lesions. The exclusion criteria included (1) active viral infections; (2) concomitant active diseases including synchronous cancer and diabetes mellitus (DM), except for DM secondary to pancreatic cancer; and (3) any prior history of biguanide use in the year before enrollment, cancer immunotherapy, and transplantation therapy.

All the study procedures were performed after written informed consent was obtained from each patient. This study was approved by the institutional review board of each participating hospital in accordance with the Declaration of Helsinki. The protocol summary, including participant and intervention details, has been described previously [18].

### Treatment scheme

Three cohorts were organized based on metformin dose level in the part 1. Metformin administration in cohort 1 was started at a dose of 750 mg/day, we had defined as a clinically reasonable dose, on days 1–15 in each cycle, increased to 1,500 (cohort 2) and 2,250 mg/day (cohort 3) with fixed dose of nivolumab (3 mg/kg) on day 1. Based on the result of DLT evaluation in the part 1, the optimal dose of metformin was selected and administered in part 2. The treatment cycle was repeated every 2 weeks, continued until disease progression or unacceptable adverse effects in both of parts.

### Toxicity assessment and dose escalation

DLT was evaluated for the first two cycles at each cohort using the Common Terminology Criteria for Adverse Events v. 4.0 (CTCAE v4.0). The MTD and the recommended doses were determined using the conventional 3 + 3 cohort method. The cohort was elevated if none of the three patients developed DLT (Table 1). However, if one of the three patients experienced a DLT, the other three patients were treated at the same dose level. The MTD was defined as a dose level that produced any DLT in ≥ two of six patients. Patients were excluded and replaced from the DLT evaluation population, in cases of without occurring DLT events (1) lower dose intensity, defined as a single dose multiplied by the number of doses of metformin actually administered during the period, compared to 70% of the planned dose intensity during the period; (2) nivolumab not administered twice in the period; and (3) insufficient patient follow-up period.

**Table 1:** Dose-limiting toxicity (DLT)

(1) Hematological toxicity
a. Grade 4 neutropenia lasting > 7 days or longer
b. Grade 4 thrombocytopenia requiring platelet transfusion
(2) Non-hematological toxicity
a. ≥ Grade 3 febrile neutropenia
b. ≥ Grade 2 hypoglycemia
c. ≥ Grade 3 other non-hematological toxicity

### PD-L1 expression

PD-L1 expression in tumor specimens, irrespective of the time when obtained, was analyzed by immunohistochemical (IHC) staining with anti-PD-L1 antibodies 28-8 and 22C3.

### Metformin pharmacokinetics and assessment for efficacy

Blood samples were collected at three points: before, and 2 h, and 4 h after metformin administration, on any day from Cycle 1 Day 8 to the day before nivolumab administration in Cycle 2 Day 1, and after confirming the stable administration of the prescribed dose of metformin for at least three consecutive days for pharmacokinetic analyses of metformin. The plasma concentrations were measured using high-performance liquid chromatography (HPLC).

Efficacy was determined by evaluating the overall response rate (ORR) according to the Response Evaluation Criteria for Solid Tumors (ver. 1.1). Progression-free survival (PFS) and overall survival (OS) were defined as the time from Cycle 1 Day 1 to the date of progressive disease or death by any cause, or until death, respectively.

### Study endpoints

The primary endpoint of part 1 is safety, evaluated in terms of the MTD and DLT, whereas the pharmacokinetics of metformin and adverse events profiling are endpoints of parts 1 and 2. The secondary endpoint was efficacy in terms of tumor shrinkage effects, PFS, and effective blood concentration of metformin. After identifying a suitable dose, an expanded assessment of safety and preliminary efficacy at the optimal dose was performed in part 2. OS was also added as a secondary endpoint after protocol amendment.

### Statistical analysis

The data from all patients who received at least one dose of study drug were included in the analysis. Statistical analysis was performed as follows according to the characteristics of the data scale. For categorical data, frequencies and rates were calculated. The Clopper–Pearson method was used to estimate confidence interval for rate. For continuous data, summary statistics were estimated. For survival time data, the Kaplan–Meier method was used to estimate cumulative survival rate, and the Greenwood method was used to estimate confidence interval. Median survival time and confidence interval were also estimated. Scatter plot, box-and-whisker plot, waterfall plot, and Kaplan–Meier curve etc. were used for graphical presentation as appropriate. Statistical hypothesis testing was not applied in this study. The confidence coefficient for estimating two-tailed confidence interval was set at 95%. Statistical data analysis was performed using SAS software (version 9.04).

## Results

### Patient characteristics and treatment delivery

In total, 41 patients with solid tumors were enrolled between August 2017 and December 2018. Patient characteristics are shown in Table 2. 41 patients comprised 24 men and 17 women, with a median age of 62 years (range, 33–76 years). Cancer types included pancreatic cancer (*n* = 26), thymic epithelial tumors, including both thymic carcinoma and invasive thymoma (*n* = 8), non-small cell lung cancer (*n* = 2), and others (*n* = 5). Other cancers included rectal, breast, esophageal, intrahepatic bile duct, and prostate cancers.

**Table 2:** Patient characteristics

	*N* = 41
Age, years	
Median (range)	62 (33–76)
Sex	
Male / Female	24 / 17
Cancer type	
Pancreatic cancer	26
Thymic epithelial tumor	8
Non-small cell lung cancer	2
Others*	5
Stage	
I / II / III / IV / Recurrence after operation or RT	1 / 4 / 24 / 12
ECOG PS	
0 / 1	19 / 22
PD-L1 status (28–8)	
< 1 / 1–49 / ≥ 50 (%) / NA	26 / 8 / 3 / 4
PD-L1 status (22C3)	
< 1 / 1–49 / ≥ 50 (%) / NA	26 / 8 / 2 / 5

^*^esophageal cancer, intrahepatic bile duct cancer, rectal cancer, breast cancer and prostate cancer (1 each)

*RT* radiation, *ECOG* Eastern Cooperative Oncology Group, *PS* performance status, *PD-L1* programmed death ligand 1, *NA* not available

**Fig. 1:**
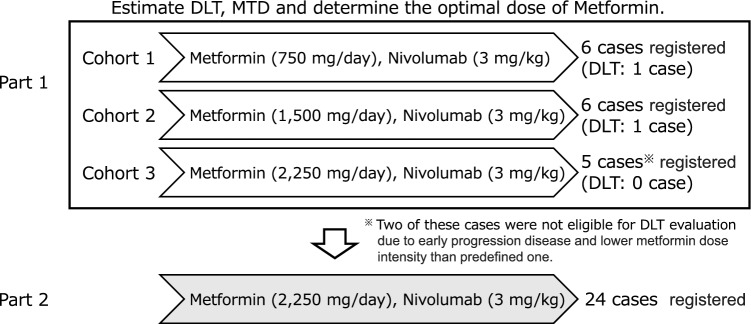
Consort diagram

Patients were administered the combination treatment in both parts 1 and 2. In part 1, six, six and five patients were treated cohort 1 (750 mg/day), 2 (1,500 mg/day), and 3 (2,250 mg/day), respectively (Fig. 1). As of August 2021, treatment was discontinued in 39 (95.1%) of the 41 patients.

### Maximum-tolerated dose in part 1

Table 3 shows the DLT evaluation in part 1. At either cohort 1 or 2, only one of the six patients developed DLT, and thus, we moved to cohort 3. Three patients were initially enrolled in cohort 3, but two were removed from DLT evaluation due to early progression disease and low metformin dose intensity of less than 70% of the defined dose, so ultimately five patients were enrolled. Since DLT was not observed in any of the patients allocated to cohort 3, MTD was not determined in part 1. Along with the recommendation by the independent data monitoring committee, the recommended dose for metformin in part 2 was determined to be 2,250 mg/day daily when co-administered with 3 mg/kg of nivolumab every 2 weeks.

**Table 3:** Dose-limiting toxicity evaluation in part 1

Event	Dose level of metformin		
	Level 1 (*N* = 6) 750 mg/day	Level 2 (*N* = 6) 1500 mg/day	Level 3 (*N* = 3) 2250 mg/day
Increased pancreatic enzymes	0	1* (16.7)	0
Abnormal liver function	0	1* (16.7)	0
pleural effusion	1 (16.7)	0	0

No. of patients (%)

^*^These events were occurred in one patient in cohort 2

### Dose intensity of nivolumab and metformin

Mean and median number of cycles for nivolumab were 10.2 ± 18.97 (standard deviation) and 5.0 (range: 1–99) cycles. No dose-dependent changes were observed at 3 mg/kg. The mean relative dose intensity of nivolumab was 96.14 ± 10.73%. Mean and median number of cycles for metformin were 9.3 ± 18.90 and 5.0 (1–98) cycles, mean and median number of dose reductions was 0.5 ± 0.68 and 0 (0–2), and mean and median number of drug suspension were 13.8 ± 39.87 and 5.0 (0–255). Mean and median relative dose intensity of metformin were 67.51±29.21% and 78.64 (3.1–103.5)%.

### Adverse events

Adverse events were observed from the start of the first administration of the study drug until 30 days after the last administration. Severe adverse events associated with this combination therapy are shown in Table 4. In general, only known adverse events were observed. Immune-related adverse events, such as hypopituitarism and adrenocorticotropin deficiency, were consistent with the adverse events associated with nivolumab. One patient developed elevated pancreatic enzyme levels (grade 4) and lactic acidosis (grade 3). No Grade 5 adverse events were observed.

**Table 4:** Severe adverse event related with this combination therapy

Event	Grade of event		
	Any	Grade 3	Grade4
Pneumonia	1 (2.4)	1 (2.4)	0
Impetigo	1 (2.4)	1 (2.4)	0
Encephalitis	1 (2.4)	0	0
Abnormal liver function	1 (2.4)	1 (2.4)	0
Hypopituitarism	1 (2.4)	1 (2.4)	0
Adrenocorticotropin deficiency	1 (2.4)	1 (2.4)	0
Pleural effusion	1 (2.4)	1 (2.4)	0
Pneumonitis	1 (2.4)	0	0
Increased pancreatic enzymes	1 (2.4)	0	1 (2.4)
Lactic acidosis	1 (2.4)	1 (2.4)	0

No. of patients (%)

(*N* = 41)

### Blood concentration of metformin and its association with safety

Blood samples were collected from 37 patients at a median of 8 days, ranging from 6 to 11 days, after administration of the first dose of the combination therapy. Both the maximal and the trough concentrations of metformin at steady state increased in a dose-dependent manner (Table 5 and Supplemental Fig. 1). At all doses, the maximum concentration increased to nearly twice the trough concentration.

**Table 5:** The blood concentration of metformin

	Dose level of metformin	N	mean (ng/mL)	SD (ng/mL)	min (ng/mL)	max (ng/mL)
*C*_trough_	Total	37	515.4	256.6	145.4	1375.5
	750 mg	6	356.2	242.2	145.4	711.9
	1500 mg	8	485.7	178.8	203.2	739.4
	2250 mg	23	567.3	272.5	244.8	1375.5
*C*_max_	Total	37	1264.9	517.5	384.7	2849.1
	750 mg	6	814.5	449.0	384.7	1642.7
	1500 mg	8	1139.0	378.4	582.6	1701.9
	2250 mg	23	1426.2	508.4	749.2	2849.1

Blood samples were collected at 3 points: before, 2 h after, and 4 h after metformin administration, on any day from Cycle 1 Day 8 to the day before nivolumab administration in Cycle 2 Day 1, after confirming the stable administration of the prescribed dose of metformin for at least 3 consecutive days

*SD* standard deviation

### Antitumor activity

An objective response was observed in 4 of 41 patients (9.8% [95% confidence interval (CI): 2.7–23.1]. (Table 6). A waterfall plot is shown in Supplemental Fig. 2. One-year PFS rate and OS rate were 9.8% [95% CI:3.1–21.0] and 56.8% [95% CI:34.2–74.3], respectively, with a median follow-up time of 5.1 months [range: 1.4–46.7] (Supplemental Fig. 3). The median PFS was 2.04 months (95% CI: 1.02–2.53]), and in patients who received the maximum application amount (MAA) from the start, the median PFS was 1.87 months (95% CI: 0.95–2.33). No association was observed between dose and PFS. ORR and 1-year OS rates were 9.1% (1 of 11) and 43.8%, and 12.0% (3 of 25) and 71.2%, in patients positive and negative for anti-PD-1 antibodies with either 28–8 or 22C3, respectively. No obvious difference in the response was evident according to metformin concentration (Table 6 and Supplemental Fig. 4).

**Table 6:** Overall response stratified by the metformin dose

			Dose level of metformin					
Response	Total (*N* = 41)		750 mg/day (*N* = 6)		1500 mg/day (*N* = 9)^※^		2250 mg/day (*N* = 26) ^※^	
	*n* (%)	95% CI	*n* (%)	95% CI	*n* (%)	95% CI	*n* (%)	95% CI
CR	0	0.0–8.6	0	0.0–45.9	0	0.0–33.6	0	0.0–13.2
PR	4 (9.8)	2.7–23.1	1 (16.7)	0.4–64.1	1 (11.1)	0.3–48.2	2 (7.7)	0.9–25.1
SD	10 (24.4)	12.4–40.3	3 (50.0)	11.8–88.2	1 (11.1)	0.3–48.2	6 (23.1)	9.0–43.6
PD	27 (65.9)	49.4–79.9	2 (33.3)	4.3–77.7	7 (77.8)	40.0–97.2	18 (69.2)	48.2–85.7
NE	0	0.0–8.6	0	0.0–45.9	0	0.0–33.6	0	0.0–13.2
ORR	4 (9.8)	2.7–23.1	1 (16.7)	0.4–64.1	1 (11.1)	0.3–48.2	2 (7.7)	0.9–25.1
DCR	14 (34.1)	20.1–50.6	4 (66.7)	22.3–95.7	2 (22.2)	2.8–60.0	8 (30.8)	14.3–51.8

^※^Of the patients who participated in part 2, three patients started with an initial dose of 1,500 mg due to the metformin reduction criteria

*CR* complete response, *PR* partial response, *SD* stable disease, *PD* progressive disease, *NE* not evaluated, *ORR* objective response rate, *DCR* disease control rate, *CI* confidence interval

ORR was 7.7% [95% CI: 0.9–25.1], and the disease control rate was 23.1% [95% CI: 9.0–43.6] when limited to pancreatic cancer, the most commonly registered cancer type. One-year PFS and OS rates were 9.8% [95% CI:3.1–21.0] and 30.1% [95% CI:7.4–57.4], respectively.

## Discussion

Part 1 of this study aimed to evaluate the safety of nivolumab metformin combination therapy in terms of maximum tolerated dose (MTD), dose-limiting toxicity (DLT), and adverse events, along with metformin pharmacokinetics, whereas Part 2 evaluated the effects of nivolumab metformin combination therapy in patients with NSCLC or thymic epithelial tumor or pancreatic cancer refractory to standard primary treatment. DLT was not observed in any of the three patients at the maximum dose cohort of 2,250 mg/day for metformin. Therefore, the recommended dose of metformin in combination with nivolumab was determined to be 2,250 mg/day. Although increased pancreatic enzyme levels were observed as grade 4 adverse events, no serious adverse event was observed. Two patients developed grade 1 hypoglycemia, and one developed grade 3 lactic acidosis, possibly related to metformin. Lactic acidosis was rated as likely associated with cancer exacerbation although the effect of metformin could not be ruled out. Overall, no apparent increase in adverse events was observed with the addition of metformin to nivolumab treatment.

An objective response was observed in 4 of 41 patients (9.8% [95% CI: 2.7–23.1]. One-year PFS and OS rates were 9.8% [3.1–21.0] and 56.8% [34.2–74.3], respectively. No correlation was observed between blood levels of metformin and treatment efficacy. Additionally, no significant differences were observed in response or survival according to tumor PD-L1 expression levels. Moreover, two patients (pancreatic cancer, 1; colorectal cancer, 1) remained alive without disease progression for more than three years. Microsatellite instability (MSI) was stable for pancreatic cancer cases; however, further testing was not possible because of the lack of tissue samples [19]. In contrast, the colorectal cancer patient was considered to have responded to ICIs due to high MSI. This is consistent with a phase II trial of nivolumab plus metformin in microsatellite-stable colorectal cancer which showed limited therapeutic benefits, with only two patients achieving stable disease. Additionally, in this phase II trial, metformin alone did not significantly alter the percentages of leukocytes and effector or central memory CD8 + T cells in patient tissues [10]. Blood levels of metformin in our study were lower than the 1.7 μg/mL that was effective in the mouse model [11], and it is possible that the oral metformin dose (2,250 mg/day) was insufficient. Higher doses of metformin might be tolerated and potentially more effective. In our study, immune biomarkers in the tumor microenvironment were not examined, and the extent to which metformin affects nivolumab was unknown.

Nonetheless, this study has some limitations. There are various reports on the efficacy of metformin against cancer, including activation of immune cells, metabolic changes in tumor cells, improvement of the immunosuppressive environment, and promotion of apoptosis of cancer cells [20]. There seems a complex mechanism for its interaction with immune checkpoint inhibitors. We did not include data on the individual effects of each drug in this trial, and thus it seems impossible to conclusively determine the presence of a synergistic effect. Without assessing the biomarkers related to immune activation or tumor metabolism, it remains unclear how metformin influences the tumor microenvironment or potentiates the effects of nivolumab in humans. Furthermore, this study could not collect information on histological sub-classification, which may be useful in the search for biomarkers. Future trials could benefit from examining optimal dosing regimens and incorporating biomarkers such as markers of mitochondrial activity or immune cell infiltration to better predict and enhance responses. Moreover, stratifying patients based on their metabolic or immunological profiles may allow for more tailored approaches.

In conclusion, this phase Ib study demonstrated that the combination of metformin and nivolumab was well-tolerated in patients with solid tumors. Thus, in future, it will be necessary to evaluate the underlying mechanism responsible for its effectiveness and conduct further studies, including phase 2 trials, with consideration given to biomarkers. Further studies including higher dose levels of metformin may be warranted. We have decided not to initiate a consecutive phase II trial and are continuing to follow up a few cases with durable responses in this phase I trial. We hope that this exploratory study will provide a new avenue for research toward the discovery of novel treatment strategies for various solid tumors.

## Supplementary Information

Below is the link to the electronic supplementary material.

Supplementary file1 (PDF 583 KB)

## Data Availability

The data that support the findings of this study are not openly available due to reasons of sensitivity and are available from the corresponding author upon reasonable request.
