# Supplementary material for: Phase-Ib dose-finding and pharmacokinetic trial of metformin combined with nivolumab for refractory/recurrent solid tumors
Source: Int J Clin Oncol. 2025 May 28;30(8):1537–44. doi: 10.1007/s10147-025-02786-2 (PMC12296837; doi:10.1007/s10147-025-02786-2)
Supplement: Supplementary file 1 — Supplementary file1 (PDF 583 KB) [file 10147_2025_2786_MOESM1_ESM.pdf]

Supplemental Figure 1

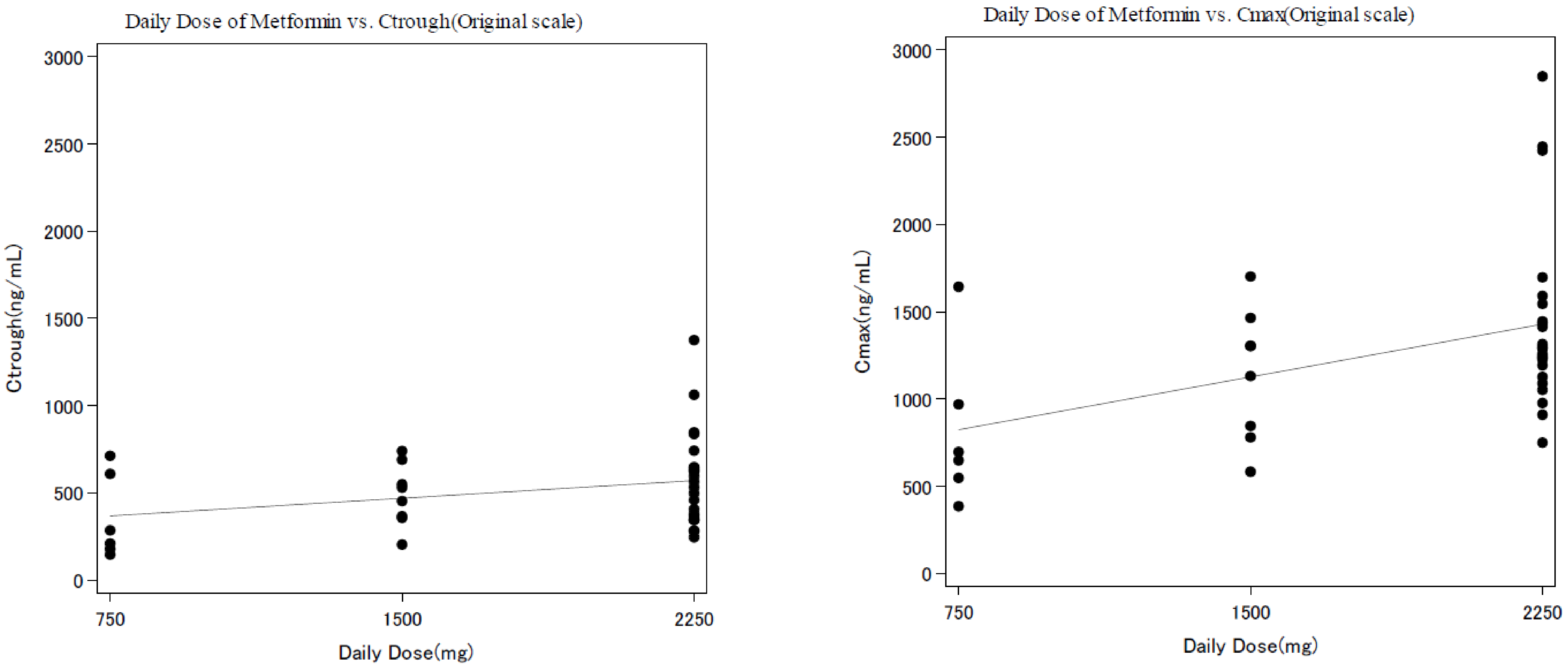

Supplemental Figure 2

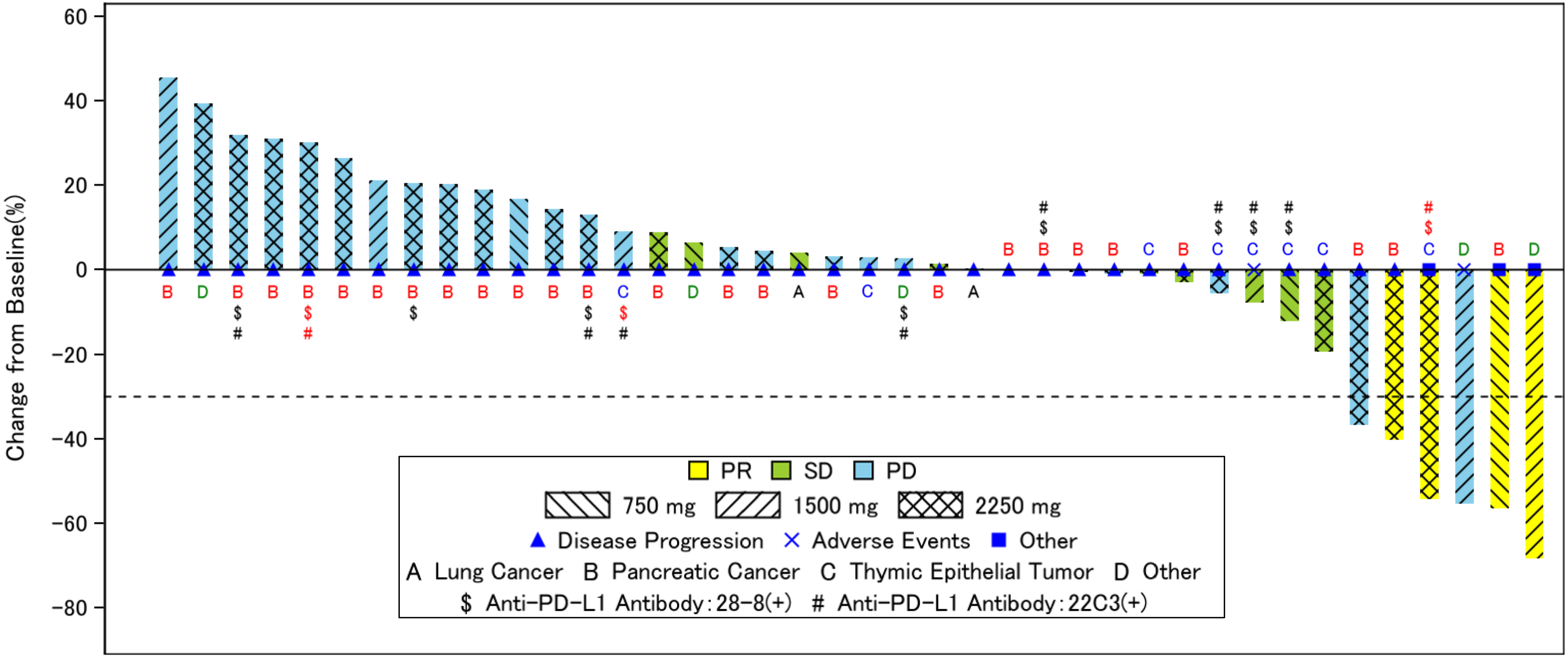

PR: partial response, SD: stable disease, PD: progressive disease
$, #: Highly positive for Anti-PD-L1 Antibody makes the symbol in red.
N=40 excluding one case of unmeasurable lesion.

Supplemental Figure 3

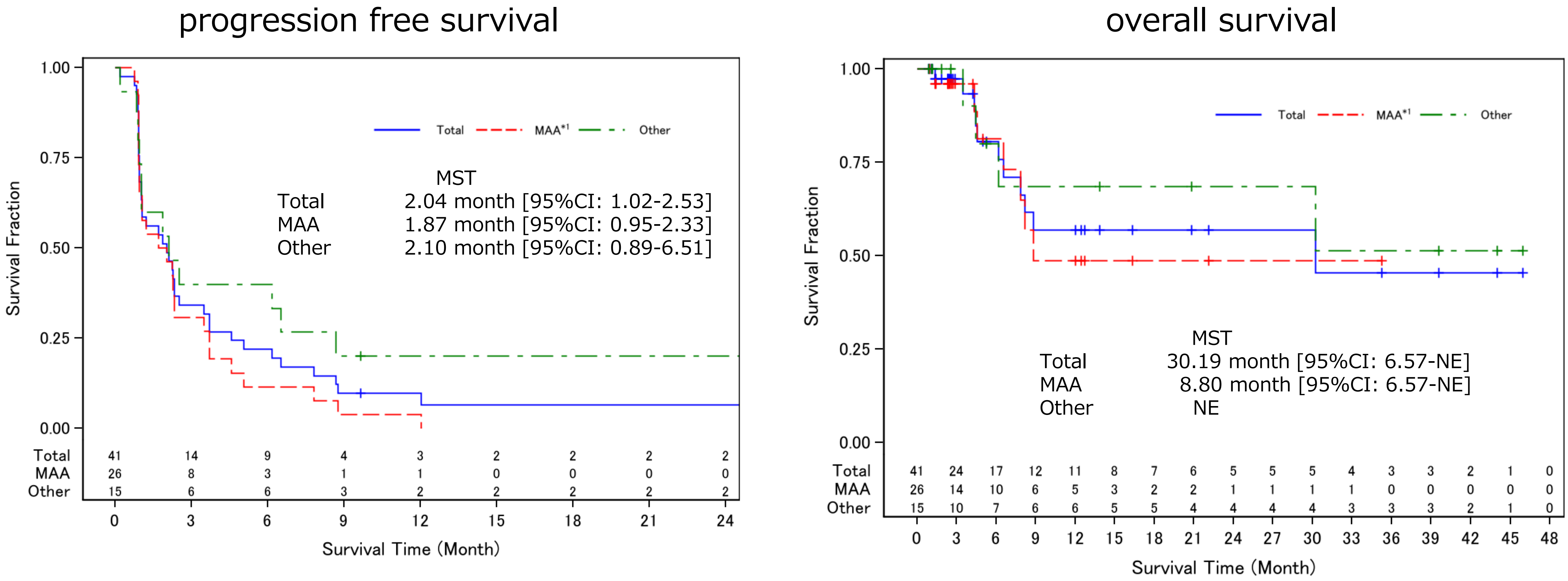

MAA: Maximum application amount, MST: median survival time, CI: confidence interval, NE: not estimated

Supplemental Figure 4

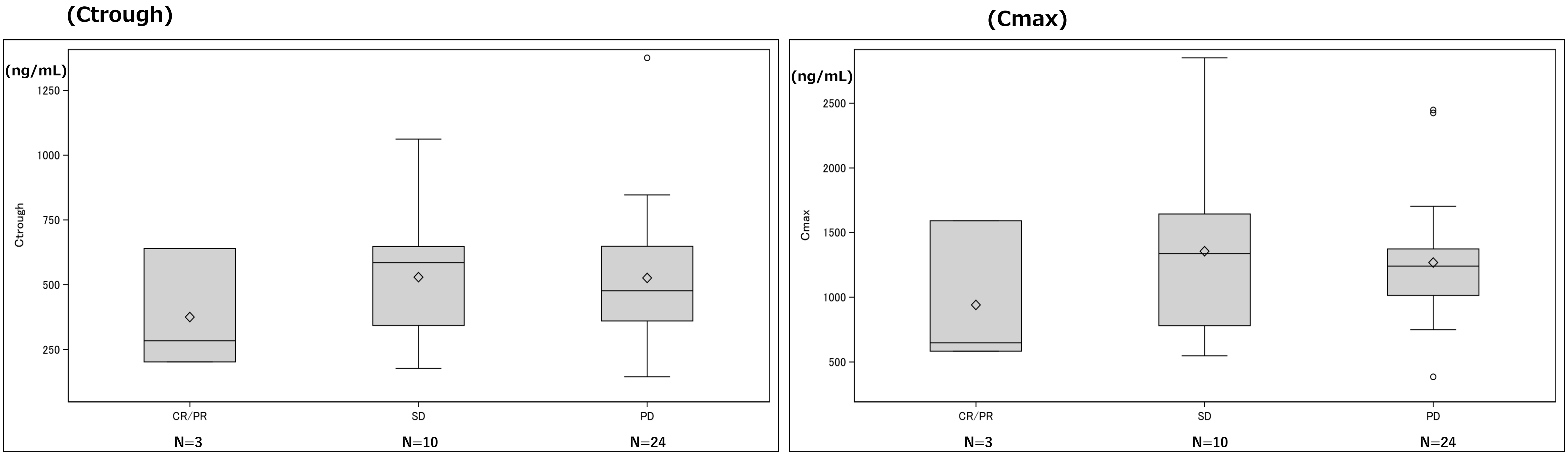

◇: mean
ORR: overall response rate
